# Broadcast Approach to Uplink NOMA: Queuing Delay Analysis

**DOI:** 10.3390/e24121757

**Published:** 2022-11-30

**Authors:** Maha Zohdy, Ali Tajer, Shlomo Shamai (Shitz)

**Affiliations:** 1 Department of Electrical, Computer, and Systems Engineering, Rensselaer Polytechnic Institute, Troy, NY 12180, USA; 2 Faculty of Electrical Engineering, Technion—Israel Institute of Technology, Haifa 3200003, Israel

**Keywords:** broadcast approach, channel state information, latency, multiple access

## Abstract

Emerging wireless technologies are envisioned to support a variety of applications that require simultaneously maintaining low latency and high reliability. Non-orthogonal multiple access techniques constitute one candidate for grant-free transmission alleviating the signaling requirements for uplink transmissions. In open-loop transmissions over fading channels, in which the transmitters do not have access to the channel state information, the existing approaches are prone to facing frequent outage events. Such outage events lead to repeated re-transmissions of the duplicate information packets, penalizing the latency. This paper proposes a multi-access broadcast approach in which each user splits its information stream into several information layers, each adapted to one possible channel state. This approach facilitates preventing outage events and improves the overall transmission latency. Based on the proposed approach, the average queuing delay of each user is analyzed for different arrival processes at each transmitter. First, for deterministic arrivals, closed-form lower and upper bounds on the average delay are characterized analytically. Secondly, for Poisson arrivals, a closed-form expression for the average delay is delineated using the Pollaczek-Khinchin formula. Based on the established bounds, the proposed approach achieves less average delay than single-layer outage approaches. Under optimal power allocation among the encoded layers, numerical evaluations demonstrate that the proposed approach significantly minimizes average sum delays compared to traditional outage approaches, especially under high arrival rates.

## 1. Introduction

There is a growing need for maintaining low latency and high reliability in a wide range of wireless communication systems [1]. Among the recently proposed techniques for attaining the latency-reliability requirements is the power domain non-orthogonal multiple access (NOMA) [2,3,4,5,6]. Uplink power domain NOMA [5] facilitates simultaneous multi-user channel access, alleviating the traditional signaling period at the beginning of the transmission. Furthermore, by leveraging power control and adaptive decoding order among users, NOMA techniques enhance user fairness by taking into consideration the dissimilarities in the channel state of each user [7,8].

A fundamental challenge that NOMA faces in wireless networks is that its power control critically relies on the availability of full channel state information at each transmitter (CSIT). This assumption is generally unfeasible under the anticipated network scale growth. In the absence of CSIT, traditional NOMA occasionally suffers from outage events, which necessitate repeated re-transmissions and negatively affect the overall latency. To address this issue, we propose a non-orthogonal multi-access technique in which each transmitter splits its stream of information into multiple encoded layers, each adapted to a specific combination of all the network’s channel states. Each user then transmits the superposition of all its encoded layers to the receiver. In particular, we approach the problem of minimizing the overall communication latency from a cross-layer resource allocation perspective by focusing on the dominant delay factor, i.e., the queuing delay [9]. The goal of the proposed approach is to minimize the average sum-queuing delay among users by optimally allocating power among the encoded layers at each transmitter in the physical layer.

Outage avoidance via multi-layer superposition coding was first proposed in [10,11] for the slowly fading single-user channels. This is generally referred to as the broadcast approach [12]. Furthermore, the studies in [13] extended the broadcast approach to the energy harvesting settings, those in [14,15,16,17,18,19,20] to random and multi-access channel models, and those in [21,22] to the multiuser interference channel. Aside from analyzing the achievable rate regions of multi-layer superposition coding [17,23], the average delay performance has only been studied for the single-user fading channel in [24]. However, under CSIT uncertainties, the advantages of adaptive multi-layer superposition coding for controlling the average queuing delay in multiple access channels are yet to be explored. Finally, we note that the broadcast approach is related to the studies on the “rate-splitting”, the foundations of which rely on superposition coding of the layered information messages [25].

In this paper, we consider an *N*-user block fading multiple access channel (MAC) in which all transmitters are oblivious to their instantaneous channel state. Each user possesses an infinite capacity queue, occasionally holding the arriving information packets to be transmitted. A novel multi-layer superposition coding scheme is then employed, in which each transmitter adapts its message to the combined network state. Based on the proposed scheme, closed-form lower and upper bounds on the average delay are characterized analytically for deterministic arrivals. Furthermore, a closed-form expression for the average queuing delay is delineated for Poisson arrivals. Based on the derived bounds on average delay, the proposed approach is shown to outperform the single-layer outage approach. Finally, under optimal power allocation among the encoded layers, numerical evaluations demonstrate that the broadcast approach significantly reduces the average sum delays compared to traditional outage approaches under symmetric/asymmetric arrival rates and channel statistics among users.

A rich literature exists on minimizing the average delay through cross-layer resource allocation in MAC with full CSIT. Relevant studies include [26] in which the authors provide an optimal solution for minimizing average delays of two-user MAC channels by controlling the departure probability of each user’s queue. In [27], an information-theoretic rate allocation policy is proposed to achieve a lower bound on the average delay of multi-access coding schemes. Dynamic power and rate control to minimize the average delay are studied for multi-access channels in [28]. The study in [28] provides a one-step value iteration policy for optimal scheduling in MAC fading channels. A lower bound on the LTE-A average delay is derived in [29] for random access channels under different arrival processes. The random access scheduling problem is addressed in [30] using a distributed virtual queue model facilitating a self-organizing policy. The study in [31] proposes a joint superposition coding and scheduling policy for the uplink NOMA by relying on user-pairing to reduce the complexity of analysis [32,33]. The accuracy of ranking users in NOMA techniques using distance-based measures versus instantaneous signal-to-noise ratio (SNR) is addressed in [34]. Joint scheduling and superposition coding in fading channels is studied in [35]. The effect of unsaturated traffic in uplink NOMA is studied in [36] using tools from queuing theory. Interaction between power control and queuing service rates in interference-limited channels is studied in [37]. Delay analysis of multi-point to multi-point networks is provided in [38] for spatial-temporal random arrival traffic. The problem of power control in delay-bounded applications is considered in [39], especially under the assumption of imperfect successive interference cancellation in uplink NOMA. The effective capacity of two-user uplink NOMA is characterized in [40] under quality-of-service delay constraints.

Energy-efficient transmission in uplink NOMA is studied in [41] under statistical delay constraints, where probabilistic upper bounds on queuing delays of NOMA are characterized. Resorting to the concept of effective capacity, the study in [42] proposes an optimized hybrid approach between non-orthogonal multiple access and orthogonal multiple access with different user pairing techniques in order to maximize the effective capacity under stringent delay constraints. Contention-based modified NOMA for uplink access is studied in [43], showing that exploiting collisions in the power domain can greatly reduce access delay. The throughput, access delay, and energy efficiency of NOMA uplink random access system are studied in [44]. Joint power control and user scheduling is considered in [45] to investigate the access delay minimization problem through an efficient sub-optimal iterative algorithm. Optimal power level partitioning to accommodate non-critical and high-priority messages is studied in [46]. A joint dynamic power control and user pairing algorithm is proposed in [47] to minimize long-term time average transmit power and queuing delay. Recent studies further includes [48] in which an adaptive rate NOMA with full CSIT is shown to provide better ergodic capacities for mobile users than OMA while satisfying strict local delay constraints for the internet of things (IoT) devices in cellular IoT networks. Opportunistic NOMA schemes are proposed in [49] for short message delivery with delay constraint based on which an upper bound on session error probability is derived, showing the impact of NOMA on session error under Rayleigh fading. A queuing delay analysis is presented in [50] for uplink NOMA with full CSIT, and the impact of channel estimation imperfections for finite-length channel coding is studied. Dynamic power allocation schemes with statistical delay quality-of-service (QoS) guarantees are shown in [51] to significantly improve the sum effective capacity and effective energy efficiency for an uplink NOMA system with paired users.

The rest of this paper is organized as follows. Section 2 presents the N-user multi-access channel model. The proposed multi-layer-based multi-access approach is outlined in Section 3 for the special case of the 2-state channel. The average delay achievable by the proposed approach is shown to outperform the average delay of the single-layer outage approach in Section 4 for deterministic and stochastic arrivals processes. The proposed multi-access approach is generalized to the case of finite arbitrary ℓ-state channel in Section 5. Finally, numerical evaluations are provided in Section 6, and the paper is concluded in Section 7.

## 2. Channel Model

Consider an *N*-user block fading MAC channel consisting of *N* transmitters and one receiver. The channel state is assumed to remain unchanged during the period of one transmission block of *n* channel uses and varies independently among consecutive blocks. We assume that the block length *n* is large enough to give rise to the notion of reliable communications but much shorter than the dynamics of the fading process [24]. Each transmitter is assumed to know the statistics of the channel state information (CSI) of its own link to the receiver but is oblivious to its instantaneous value. Complete CSI of all links is assumed to be available at the receiver. The input-output relationship of this channel is given by

(1) $$\begin{array}{c} Y=\sum_{i=1}^{N}h_{i}X_{i}+W\;, \end{array}$$

where $X_{i}$ denotes the transmitted signal from user *i* and *W* is the additive white Gaussian noise with zero mean and unit variance. Finally, $h_{i}$ denotes the state of the fading channel between transmitter *i* and the receiver. The transmitted signal $X_{i}$ is subject to an average power constraint *P* for all i∈{1,…,N}, i.e., $E\left[|X_{i}{|}^{2}\right]\leq P$. We consider a quantized model for the fading channel according to which $h_{i}^{2}$ takes one of two possible states, referred to as {*weak*, *strong*}, denoted by $\left\{\alpha_{1},\alpha_{2}\right\}$, respectively. Without loss of generality, we assume $0<\alpha_{1}<\alpha_{2}<+\infty$. User *i* experiences *strong* or *weak* channel states with probabilities $p_{i}\overset{▵}{=}P\left(h_{i}^{2}=\alpha_{2}\right)$ and ${\bar{p}}_{i}\overset{▵}{=}1-p_{i}$, respectively.

Each transmitter is assumed to possess an infinite-capacity queue. The queue at transmitter *i* receives random packets with an average arrival rate $\lambda_{i}$ (bits/channel use). The size of the data queued at transmitter *i* at the beginning of any transmission block *t* is denoted by ${\tilde{Q}}_{i}\left(t\right),\;\forall i\in \left\{1,\ldots ,N\right\}$. We define $A_{i}\left(t\right)$ as the total number of bits arriving in the queue at transmitter *i* during transmission block *t*. Finally, $r_{i}\left(t\right)$ (bits/channel use) denotes the service rate of the queue at transmitter *i*. Hence, the queue size at transmitter *i* at the end of any transmission block can be expressed using a recursive relationship as

(2) $$\begin{array}{cc} {\tilde{Q}}_{i}\left(t+1\right) & =\left\{\begin{array}{cc} {\tilde{Q}}_{i}\left(t\right)+nA_{i}\left(t\right)-nr_{i}\left(t\right), & {\tilde{Q}}_{i}\left(t\right)+nA_{i}\left(t\right)-nr_{i}\left(t\right)\geq 0\\ 0, & \mathrm{otherwise} \end{array}\right.\;. \end{array}$$

Accordingly, we define $Q_{i}\left(t\right)$ as queue size normalized by the number of transmission blocks *n*, i.e.,

(3) $$\begin{array}{cc} Q_{i}\left(t+1\right) & \overset{▵}{=}\left\{\begin{array}{cc} Q_{i}\left(t\right)+Z_{i}\left(t\right), & \;Q_{i}\left(t\right)+Z_{i}\left(t\right)\geq 0\\ 0, & \;o.w. \end{array}\right.\;, \end{array}$$

where the random variable $Z_{i}\left(t\right)$ is defined as $Z_{i}\left(t\right)\overset{▵}{=}A_{i}\left(t\right)-r_{i}\left(t\right)$, and it captures the change in the queue size at transmitter *i* at the end of transmission block *t*. We remark that the number of bit arrivals $A_{i}\left(t\right)$ is random and does not necessarily fit into the exact size of the transmitted packet in a given transmission block. Therefore, if the backlogged data at any queue is less than a packet length, the data bits are zero-padded to form a complete packet for the encoder at each transmitter. Throughout the rest of the paper, we assume that the processing delay, i.e., encoding and decoding processes, as well as the transmission delay, are fixed and negligible with respect to the queuing delay. We use the concise notation $C\left(x,y\right)\overset{▵}{=}\frac{1}{2}\log_{2}\left(1+\frac{x}{\frac{1}{P}+y}\right)$, ${\left\{x_{j}^{i}\right\}}_{j=1}^{k}\overset{▵}{=}\left\{x_{1}^{i},x_{2}^{i},\ldots ,x_{k}^{i}\right\}$. Finally, we denote the set of all users in the network by $N\overset{▵}{=}\left\{1,\ldots ,N\right\}$.

## 3. 2-State Channel Multi-Access

In this section, we present a non-orthogonal multiple-access approach based on multi-layer encoding at each transmitter and successive interference cancellation (SIC) at the receiver. The underlying layering approach hinges on adapting the number of encoded layers at each transmitter to the combined fading state of the network, i.e., the fading states of all transmitters to the receiver. Owing to the arising interference in non-orthogonal multi-access channels with no CSIT, the channel state of each user directly affects the decoding success probabilities of all the other users. Motivated by this, the recent work in [17] proposed a multi-layer coding approach for the two-user multiple access channel with no CSIT, specially adapted to the combined network state resulting in an enlarged average achievable rate regions compared to the existing multi-layer coding approaches. In this section, we extend the layering approach in [17] to the general case of an arbitrary number of *N*-users. As shown in this paper, the proposed multi-access approach enjoys considerable advantages in reducing the queuing delay.

### 3.1. Layering Approach

At the beginning of each transmission block, user *i* aims to transmit all the data bits accumulated in its queue if the channel state allows it. Otherwise, it encodes a part of its data with the maximum allowable encoding rate. Towards this goal, user *i* encodes its data (fully or partially) using 2N independent messages generated from 2N Gaussian codebooks. These messages are denoted by $U_{jk}^{i},\forall i\in N,j\in \left\{1,2\right\},k\in \left\{0\cup N\right\}$. Based on this decomposition

(4) $$\begin{array}{c} X_{i}=\sum_{j=1}^{2}\sum_{k=0}^{N}U_{jk}^{i}\;. \end{array}$$

We consider an ordering of the network states based on the number of users with *strong* channel states denoted by *k*. We define $S_{k}$ as the set of *k* users’ indices that experience *strong* channel states. Accordingly, $E_{k}$ denotes the event that exactly *k* users are experiencing a *strong* channel including.

The notation $U_{jk}^{i}$ can be interpreted as follows. Superscript *i* denotes the user index i∈N, subscript j∈{1,2} refers to user *i*’s channel state, where j=1 if $h_{i}^{2}=\alpha_{1}$ and otherwise j=2. Finally, k∈{0∪N} represents the number of users in the network with a *strong* channel state, possibly including user *i*’s channel. Therefore, for every value of *k*, user *i* adapts the rate of two codewords, ${\left\{U_{jk}^{i}\right\}}_{j=1}^{2}$, based on its own channel state resulting in a total of 2k layers. The correspondence between each channel state and the adapted layer is shown in Table 1 and summarized below:

- $U_{10}^{i}$ is adapted to $E_{0}$, where all channels are *weak*.
- $U_{2N}^{i}$ is adapted to $E_{N}$, where all channels are *strong*.
- When exactly *k* channels are *strong*:
  - $U_{1k}^{i}$ is adapted to $N\backslash E_{k}$ if user *i*’s channel is *weak*.
  - $U_{2k}^{i}$ is adapted to $E_{k}$ if user *i*’s channel is *strong*.

The rate of codeword $U_{jk}^{i}$ is denoted by $R_{jk}^{i}$. Finally, we define $\beta_{jk}^{i}$ as the power fraction of the total power *P* allocated to codeword $U_{jk}^{i}$, such that

$$\sum_{j=1}^{2}\sum_{k=0}^{N}\beta_{jk}^{i}=1\;.$$

For user *i*, the rate of each codebook is governed via the power allocation parameters $\beta_{jk}^{i}$ such that at least one layer is successfully decoded in every possible network state.

### 3.2. Decoding Approach

Corresponding to the layering approach in Section 3.1, we propose a decoding algorithm with 2kN SIC stages for each combined channel state with *k* strong channels. The layers’ decoding order is adapted to the combined channel states such that all the layers adapted to channel states with less than *k* strong users, $\left\{U_{j\ell }^{i},\forall j\in \left\{1,2\right\},\ell <k\right\}$, are first decoded and subtracted from the received signal. Afterwards, layers adapted to channel state with exactly *k* strong users, $\left\{U_{jk}^{i},\forall j\in \left\{1,2\right\}\right\}$, are decoded.

When |S|=k, the receiver employs 4k+1 decoding stages. Each of the layers for any j∈{1,2} and ℓ∈{0,…,k}, the set of codebooks $\left\{U_{j\ell }^{i}:i\in N\right\}$ is partitioned to two sets

(5) $$\begin{array}{c} P_{j\ell }≜\left\{U_{j\ell }^{i}:i\in S\right\}\;\mathrm{and}\;Q_{j\ell }≜\left\{U_{j\ell }^{i}:i\notin S\right\}\;, \end{array}$$

rendering a total of 4k+1 partitions for different j∈{1,2} and ℓ∈{0,…,k}. The decoding strategy decodes one message from each of these, except for the partition $\left\{U_{2k}^{i}:i\notin S\right\}$. The decoding strategy works as follows. We create the following two sequences of sets:

(6) $$\begin{array}{cc} P & ≜\{P_{10},P_{11},P_{21},\ldots ,P_{2(k-1)},P_{1k},\}\;, \end{array}$$

(7) $$\begin{array}{cc} Q & ≜\{Q_{1k},Q_{2(k-1)},Q_{1(k-1)},\ldots ,Q_{11},Q_{10},\}\;. \end{array}$$

The decoding strategy selects codebooks by alternating between P and Q in ascending order and decodes exactly one codebook from each. Specifically, the codebook sets are selected in the following order: $\left\{P_{10},Q_{1k},P_{11},Q_{2(k-1)},P_{21},\ldots ,P_{1k},Q_{10}\right\}$. This results in 4k coding stages. Finally, the codebooks in $\left\{U_{2k}^{i}:i\in S\right\}$ are decoded as the last stage, i.e., stage 4k+1. Next, we describe the decoding stages and the set of codebooks decoded in each.

- **Decoding stage 1:** We start by decoding the layers $P_{10}≜\left\{U_{10}^{i}:i\in S\right\}$, i.e., the codebooks $U_{10}^{i}$ of only the *k* strong users in S. We define $S_{k}$ as an ordered set of these users, in which the users are ordered in an ascending order based on their indices. The codebooks will be decoded sequentially in this order.
- **Decoding stage 2:** Next, after decoding and removing the codebooks in $P_{10}$, we sequentially decode the layers in $Q_{1k}=\left\{U_{1k}^{i}:i\notin S\right\}$, which involves layers $U_{1k}^{i}$ of users with weak channels.
- **Decoding stage 3:** In the third stage, the codebooks in $P_{10}$ and $Q_{1k}$ are already decoded. We continue by sequentially decoding the set of codebooks in $P_{11}≜\left\{U_{11}^{i}:i\in S\right\}$.
- **Decoding stage 4:** The decoding process continues by sequentially decoding the codebooks in $Q_{2(k-1)}=\left\{U_{2(k-1)}^{i}:i\notin S\right\}$, while the codebooks of $P_{10}$, $Q_{1k}$, and $P_{11}$ are already decoded.
- **Decoding stage 5:** This stage sequentially decodes the codebooks $P_{21}$.
- **Decoding stage 6:** This stage sequentially decodes the codebooks in $Q_{1(k-1)}$.
- **Decoding stages {2,…,4k+1}:** Following the pattern of the previous decoding stages, in general, in stage {2,…,4k}, we decode the codebooks according to the following schedule for ℓ∈{1,…,k}:
  (8) $$\begin{array}{c} \begin{array}{cc} \mathrm{codebooks}\mathrm{in}Q_{1(k-\ell +1)} & \;\mathrm{stage}\;4\ell -2\\ \mathrm{codebooks}\mathrm{in}P_{1\ell } & \;\mathrm{stage}\;4\ell -1\\ \mathrm{codebooks}\mathrm{in}Q_{2(k-\ell )} & \;\mathrm{stage}\;4\ell \\ \mathrm{codebooks}\mathrm{in}P_{2\ell } & \;\mathrm{stage}\;4\ell +1 \end{array} \end{array}$$

The proposed decoding approach results in decoding more layers for a channel state with *k* strong users compared to a state with k−1 strong users. In particular, the receiver decodes one extra layer for user *i* in channel state $E_{k}$ as compared to state $E_{k-1}$. Note that in both states, user *i* experiences a *weak* channel. On the other hand, the receiver decodes two extra layers for user *i* in channel state $E_{k}$ as compared to state $E_{k-1}$, note that user *i* experiences a *strong* channel in both states. Our intuition behind such a strategy hinges on two factors. First, that decoding and removing additional interfering users with strong channel states is expected to increase the achievable rate of user *i*. Secondly, when user *i* experiences a stronger channel, the receiver can possibly decode an additional layer from its message. The decoded layers for channel state $E_{k}$ are shown in Table 2 for illustration.

Finally, the detailed steps of the proposed successive decoding algorithm are presented in Algorithm 1. We remark that the effect of the precedence of users with similar channel states within each decoding stage on the average achievable delay will be analyzed in the subsequent sections.
**Algorithm 1:** Successive Decoding for 2-state channel1:**input**$\left(h_{1}^{2},\ldots ,h_{N}^{2}\right)$, *k*2:**for**ℓ∈{0,…,k}3:   **if** ℓ=04:      In stage 1 successively decode ${\left\{U_{10}^{i}\right\}}_{i=1}^{N}$5:   **else if** ℓ∈{1,…,k}6:      (1) In stage 4ℓ−2 successively decode $Q_{1(k-\ell +1)}$7:      (2) In stage 4ℓ−1 successively decode $P_{1\ell }$8:      (3) In stage 4ℓ successively decode $Q_{2(k-\ell )}$9:      (4) In stage 4ℓ+1 successively decode $P_{2\ell }$10:   **end if**11:**end for**

Based on the multi-access approach outlined throughout this section, the service rate of the queue at transmitter *i* is determined by the total rates of the successfully decoded layers during each network state. Therefore, the service rate $r_{i}\left(t\right)$ during transmission block *t* varies randomly and is jointly determined by the states of all users as well as the power allocation among different layers at each transmitter, i.e., $\beta_{jk}^{i}$. The achievable rates for all the encoded layers are formally stated in the Theorem 1.

**Theorem** **1.** *For the N-user MAC channel without CSIT, when exactly k∈N∪{0} users have strong channels, the achievable rates of the layering approach in Section 3.1 and the decoding policy in Algorithm 1 are characterized by the set of rates $\left\{R_{jk}^{i},\forall j\in \left\{1,2\right\},i\in N,\ell \in \left\{0\cup N\right\}\right\}$ that satisfy*

(9) $$\begin{array}{cc} R_{j\ell }^{i} & \leq \min_{S:|S|=k}d_{j\ell }^{i}\left(S\right)\;, \end{array}$$

*where constants $\left\{d_{jk}^{i}\left(S\right),\forall k\in \left\{0\cup N\right\},j\in \left\{1,2\right\}\right\}$ are defined in Appendix A.*

**Proof.**  See Appendix B. □

We remark that characterizing the achievable rate region of the proposed approach in the form of rate bounds on individual codebooks rates, rather than an average achievable rate region, will be instrumental to characterizing the average achievable delay analysis throughout the next section.

## 4. Average Queuing Delay

In this section, we investigate the average queuing delay achieved by the multi-access approach in Section 3 compared to the conventional single-layer (outage) multi-access approach. First, in Section 4.1, we focus on the case of the deterministic arrival process at each queue, for which we delineate lower and upper bounds on the average queuing delay. Furthermore, the case of stochastic arrivals is examined in Section 4.2 in which a closed-form expression for the average delay achievable by the proposed approach is characterized and compared to that of the single-layer transmission approach. To proceed, we define $E_{k}^{i}$ as the event in which we have exactly *k* strong channels and they include the channel of user *i*. Accordingly, we define ${\bar{E}}_{k}^{i}≜N\backslash E_{k}^{i}$. We begin by computing the probabilities of the events $E_{k}^{i}$ (and $E({\bar{S}}_{k}^{i}))$ as follows.

(10) $$\begin{array}{cc} P\left[E_{k}^{i}\right] & =\sum_{\begin{array}{c} I\subseteq N\\ \left|I\right|=k \end{array}}\prod_{j\in I}p_{j}\prod_{\begin{array}{c} \ell \notin I\\ \ell \neq i \end{array}}{\bar{p}}_{\ell }\;\;\mathrm{and}\;P\left[{\bar{E}}_{k}^{i}\right]=\sum_{\begin{array}{c} I\subseteq N\\ \left|I\right|=k \end{array}}\prod_{\begin{array}{c} j\in I\\ j\neq i \end{array}}p_{j}\prod_{\ell \notin I}{\bar{p}}_{\ell }\;. \end{array}$$

where I denotes a subset of user indices.

### 4.1. Deterministic Arrivals

Throughout this subsection, we assume that the data arrival process at each queue is a deterministic process with an average arrival rate $\lambda_{i}$, i.e., $A_{i}\left(t\right)=\lambda_{i},\;\forall i\in N$. Note that as a result of the zero-padding applied by the encoder, whenever the available data bits are fewer than a transmission packet, a G/G/1 queuing model is generated at each transmitter. A closed-form expression characterizing the average delay of the G/G/1 queuing model is, in general, unknown. Therefore we resort to characterizing upper and lower bounds on the average queuing delay. These bounds are formally presented in Theorem 2. Before stating Theorem 2, we provide an outline of the main steps pertinent to deriving the characterized bounds, where the detailed proof can be found in Appendix C.

Establishing the desired bounds hinges on characterizing the average queue size at each transmitter *i* using the Laplace transform of the probability distribution function (PDF) of the queue size $Q_{i}$ (moment generating function). Let the PDF of $Q_{i}$ be denoted by $dF_{i}\left(q\right)$ and its associated Laplace transform be denoted by $L_{i}\left(s\right)$. Therefore, the average queue size at transmitter *i* is given by

(11) $$\begin{array}{c} E\left[Q_{i}\right]=\lim_{s\to 0}-\frac{dL_{i}\left(s\right)}{ds}\;. \end{array}$$

Recalling the recursive expression for $Q_{i}$ in terms of the variable $Z_{i}$ in (3), a recursive form of $F_{i}\left(q\right)$ can be expressed as follows [52,53]

(12) $$\begin{array}{c} F_{i}\left(q\right)=\left\{\begin{array}{cc} \int_{-\infty }^{q}F_{i}\left(q-\tau \right)dF_{Z_{i}}\left(\tau \right)\;, & \;q\geq 0\;\\ 0\;, & \;q<0 \end{array}\right.\;, \end{array}$$

where $dF_{Z_{i}}\left(z\right)$ denote PDF of $Z_{i}$ denoting change in queue size at user *i*. At the end of every transmission block, the change in queue size *i*, $Z_{i}$, is primarily determined by the difference between the data arrival $\lambda_{i}$ and the total rate of all the layers successfully decoded by the receiver from user *i*’s message stream, which in turn is determined by the combined network state. Consequently, $dF_{Z_{i}}\left(z\right)$ can be expressed as

(13) $$\begin{array}{cc} dF_{Z_{i}}\left(z\right) & =P\left[E\left({\bar{S}}_{0}^{i}\right)\right]\delta \left(z-\lambda_{i}+R_{10}^{i}\right)+P\left[E\left(S_{N}^{i}\right)\right]\delta \left(z-\lambda_{i}+\sum_{j=1}^{2}\sum_{k=1}^{N}R_{jk}^{i}\right)\\ \; & +\sum_{\ell =1}^{N-1}P\left[E\left(S_{\ell }^{i}\right)\right]\delta \left(z-\lambda_{i}+\sum_{j=1}^{2}\sum_{k=0}^{\ell -1}R_{jk}^{i}+R_{2\ell }^{i}\right)\\ \; & +\sum_{\ell =1}^{N-1}P\left[E\left({\bar{S}}_{\ell }^{i}\right)\right]\delta \left(z-\lambda_{i}+\sum_{j=1}^{2}\sum_{k=0}^{\ell -1}R_{jk}^{i}+R_{1\ell }^{i}\right)\;. \end{array}$$

We remark that in order to guarantee the stability of the data queue at each transmitter, we assume that the arrival rate $\lambda_{i}$ is less than the average achievable rate (service rate of the queue), i.e.,

(14) $$\begin{array}{c} \lambda_{i}<E\left[r_{i}\right]\;,\;\forall i\in N\;, \end{array}$$

where the average service rate at queue *i* is given by

(15) $$\begin{array}{cc} E[r_{i}] & =P\left[E\left({\bar{S}}_{0}^{i}\right)\right]R_{10}^{i}+P\left[E\left(S_{N}^{i}\right)\right]\cdot \sum_{j=1}^{2}\sum_{k=1}^{N}R_{jk}^{i}\\ \; & +\sum_{\ell =1}^{N-1}P\left[E\left(S_{\ell }^{i}\right)\right]\cdot \left(\sum_{j=1}^{2}\sum_{k=0}^{\ell -1}R_{jk}^{i}+R_{2\ell }^{i}\right)+\sum_{\ell =1}^{N-1}P\left[E\left({\bar{S}}_{\ell }^{i}\right)\right]\cdot \left(\sum_{j=1}^{2}\sum_{k=0}^{\ell -1}R_{jk}^{i}+R_{1\ell }^{i}\right)\;. \end{array}$$

An explicit expression for $F_{i}\left(q\right),\forall i\in N$, directly follows by combining (12) and (13)

(16) $$\begin{array}{cc} \; & F_{i}\left(q\right)=\left\{\begin{array}{cc} 0\;, & \;\forall q\in R_{1}\\ P\left[E\left(S_{0}^{i}\right)\right]F_{i}\left(q-\lambda_{i}+\sum_{j=1}^{2}\sum_{k=0}^{N}R_{jk}^{i}\right)\;, & \;\forall q\in R_{2}\\ \;\vdots \\ P\left[E\left({\bar{S}}_{0}^{i}\right)\right]F_{i}\left(q-\lambda_{i}+R_{10}^{i}\right)\;, & \;\forall q\in R_{2N-1} \end{array}\right.\;, \end{array}$$

where the intervals $R_{i},\forall i\in \left\{1,\ldots ,2N-1\right\}$, are given by

$$\begin{array}{cc} R_{1} & \overset{▵}{=}\left(-\infty ,0\right)\;,\\ R_{2} & \overset{▵}{=}\left[0,\lambda_{i}-\sum_{j=1}^{2}\sum_{k=0}^{N}R_{jk}^{i}+R_{1(N-1)}^{i}\right]\;,\\ \; & \vdots \\ R_{2N-1} & \overset{▵}{=}\left[\lambda_{i}-R_{10}^{i},\infty \right)\;. \end{array}$$

Finally, the Laplace transform of the queue size PDF is computed using (16), which in turn facilitates obtaining the average queue size at user *i*. Note that although $F_{i}\left(q\right)$ is expressed in (16), it is still a recursive form. Therefore, the obtained expression for the average queue size delay contains the unknown term $F_{i}\left(q\right)$, which is why a closed form cannot be obtained. Subsequently, an upper and a lower bound on the average queue size of user i∈N are formally characterized in the next theorem.

**Theorem** **2.** *The average queue size of transmitter i under the multi-access policy in Section 3 is bounded by*

(17) $$\begin{array}{c} \frac{1}{2}\sum_{j=1}^{2}\sum_{k=0}^{N}R_{jk}^{i}-\frac{\lambda_{i}}{2}-\frac{N_{i}}{D_{i}}\leq E\left[Q_{i}\right]\leq \sum_{j=1}^{2}\sum_{k=0}^{N}R_{jk}^{i}-\lambda_{i}-\frac{N_{i}}{D_{i}}\;, \end{array}$$

*where we have defined $D_{i}\overset{▵}{=}2\left(E\left[r_{i}\right]-\lambda_{i}\right)$ and*

(18) $$\begin{array}{cc} \; & N_{i}\overset{▵}{=}-{\left(\sum_{j=1}^{2}\sum_{k=0}^{N}R_{jk}^{i}-\lambda_{i}\right)}^{2}+P\left[E\left({\bar{S}}_{0}^{i}\right)\right]{\left(\sum_{j=1}^{2}\sum_{k=1}^{N}R_{jk}^{i}\right)}^{2}\\ \; & +\sum_{\ell =1}^{N-1}P\left[E\left(S_{\ell }^{i}\right)\right]\cdot {\left(\sum_{j=1}^{2}\sum_{k=\ell +1}^{N}R_{jk}^{i}+R_{1\ell }^{i}\right)}^{2}+\sum_{\ell =1}^{N-1}P\left[E\left({\bar{S^{i}}}_{\ell }\right)\right]\cdot {\left(\sum_{j=1}^{2}\sum_{k=\ell +1}^{N}R_{jk}^{i}+R_{2\ell }^{i}\right)}^{2}\;. \end{array}$$

**Proof.**  See Appendix C. □

Using Little’s law, upper and lower bounds on the average queuing delay at transmitter *i* under deterministic arrivals can directly be obtained by normalizing the bounds characterized in Theorem 2 $E[Q_{i}]$ by $\lambda_{i}$.

In order to assess the performance of the proposed multi-layer superposition coding access approach, we compare the achievable average queuing delay to that of the conventional single-layer access (outage) approach. To this end, we first summarize the single-layer approach, and afterward, a lower bound on the average queuing delay achieved by the single-layer approach is characterized in Lemma 1. Finally, we compare the rate of increase of the average delay achieved by each policy with respect to the data arrival rate. As the arrival rate increases, the rate of increase of the average delay with respect to $\lambda_{i}$ resulting from the proposed approach is lower than that resulting from the single-layer (outage) approach.

According to the single-layer (outage) transmission approach, each transmitter encodes the available data in its queue into one layer of a fixed rate irrespective of the unknown network state. For i∈N, let $R_{i}^{s}$ denote the rate of the single encoded layer transmitted by user *i* in the outage approach. In any given transmission block, if the rate $R_{i}^{s}$ lies in the achievable rate region of the actual network state, it will be successively decoded by the receiver. Otherwise, an outage occurs where the receiver fails to decode the message of user *i*, and the transmitter attempts to re-transmit the same message in the subsequent transmission block using the same encoding rate $R_{i}^{s}$. We define $r_{i}^{s}\left(t\right)$ as the service rate of the queue at user *i* under the single-layer transmission, the encoding rate of the codeword transmitted by user *i* in transmission block *t* and successively decoded by the receiver, hence removed from user *i*’s queue. Furthermore, we denote by $p_{i}^{s}$ the probability of successfully decoding a message of rate $R_{i}^{s}$ from user *i*. Accordingly, the service rate of the queue at transmitter *i* using the outage approach is given by

(19) $$\begin{array}{cc} r_{i}^{s}\left(t\right) & =\left\{\begin{array}{cc} R_{i}^{s},\; & \mathrm{with}\;\mathrm{probability}\;p_{i}^{s}\\ 0,\; & \mathrm{with}\;\mathrm{probability}\;1-p_{i}^{s} \end{array}\right.\;. \end{array}$$

Finally, we define $Q_{i}^{s}$ as the queuing size at transmitter *i* under the single-layer transmission approach summarized above. In Lemma 1, we characterize lower and upper bounds on the average $E[Q_{i}^{s}]$ using an approach similar to that used to characterize the bounds in Theorem 2.

**Lemma** **1.** *The average queue size of transmitter i under single layer (outage) approach is lower and upper bounded according to:*

(20) $$\begin{array}{c} \frac{1}{2}R_{i}^{s}-\frac{\lambda_{i}}{2}-\frac{{\left(R_{i}^{s}-\lambda_{i}\right)}^{2}-R_{i}^{s}\left(1-p_{i}^{s}\right)}{2\left(p_{i}^{s}R_{i}^{s}-\lambda_{i}\right)}\leq E\left[Q_{i}^{s}\right]\leq R_{i}^{s}-\lambda_{i}-\frac{{\left(R_{i}^{s}-\lambda_{i}\right)}^{2}-R_{i}^{s}\left(1-p_{i}^{s}\right)}{2\left(p_{i}^{s}R_{i}^{s}-\lambda_{i}\right)}\;. \end{array}$$

**Proof.**  Follows the same argument as that in Appendix C. □

In Theorem 2 and Lemma 1, we remark that the characterized bounds on the average queuing delay at each transmitter depend only on the arrival rate at the same node. Therefore, the effect of the average arrival rate on the delay bounds in (17) or (20) can be analyzed for each node *i* independently. In Theorem 3, while fixing the average achievable rates at each user among both approaches, we show that as the arrival rate $\lambda_{i}$ at each user increases, the proposed multi-access approach lower rate of increase in the average queuing delay with respect to that achieved by the single layer approach.

**Theorem** **3.** *For the N-user multiple access channel, given that*

(21) $$\begin{array}{c} E\left[r_{i}\right]=E\left[r_{i}^{s}\right]\;, \end{array}$$

*the rate of increase of average delay with respect the arrival rate under the approach in Section 3 is lower than that achieved by single-layer outage approach, i.e., for every i∈N*

(22) $$\begin{array}{c} \frac{\partial E[Q_{i}]}{\partial \lambda_{i}}\leq \frac{\partial E[Q_{i}^{s}]}{\partial \lambda_{i}}\;. \end{array}$$

**Proof.**  See Appendix D. □

### 4.2. Stochastic Arrivals

In this section, we consider the proposed multi-layer superposition coding policy presented in Section 3 under Poisson distributed random arrivals $A_{i}∼Pois\left(\lambda_{i}\right)$. We adopt the same queuing model in which each transmitter applies zero-padding in case the available bits in its queue are fewer than the size of a transmitted packet. Therefore, under Poisson distributed arrivals, the considered model constitutes an M/G/1 queuing model with an average arrival rate $\lambda_{i}$ and service rate $r_{i}$ specified in (15). Furthermore, we denote the queue utilization at transmitter *i* by $\rho_{i}\overset{▵}{=}\frac{\lambda_{i}}{E[r_{i}]}$. The average queue length for an M/G/1 queue can be characterized in a closed form by directly applying the Pollaczek-Khinchin formula. Theorem 4 formally states the average queuing size under the proposed layering and decoding approach.

**Theorem** **4.** *According to the multi-access approach outlined in Section 3, the average queue length at user i with Poisson distributed arrivals with the average rate $\lambda_{i}$ is given by*

(23) $$E\left[Q_{i}\right]=\rho_{i}+\frac{\rho_{i}^{2}+\lambda_{i}V\left[r_{i}\right]}{2(1-\rho_{i})}\;,$$

*where the average service rate $E[r_{i}]$ is given by (15) and the variance of the service rate $V[r_{i}]$ is*

(24) $$\begin{array}{cc} V[r_{i}] & =-E\left[r_{i}\right]+P\left[E\left({\bar{S}}_{0}^{i}\right)\right]{\left(R_{10}^{i}\right)}^{2}+P\left[E\left(S_{N}^{i}\right)\right]\cdot {\left(\sum_{j=1}^{2}\sum_{k=1}^{N}R_{jk}^{i}\right)}^{2}\\ \; & +\sum_{\ell =1}^{N-1}P\left[E\left(S_{\ell }^{i}\right)\right]\cdot {\left(\sum_{j=1}^{2}\sum_{k=0}^{\ell -1}R_{jk}^{i}+R_{2\ell }^{i}\right)}^{2}+\sum_{\ell =1}^{N-1}P\left[E\left({\bar{S^{i}}}_{\ell }\right)\right]\cdot {\left(\sum_{j=1}^{2}\sum_{k=0}^{\ell -1}R_{jk}^{i}+R_{1\ell }^{i}\right)}^{2}\;. \end{array}$$

**Proof.**  Follows by applying Pollaczek-Khinchin formula for the M/G/1 average queue size [54], where the service rate of queue *i* is given by $r_{i}$. □

We remark that the proof of Theorem 3 implies that the proposed approach outperforms the single-layer outage approach in the case of Poisson arrivals as well, under equal average achievable rates. This result can be readily verified given that the proof in Appendix D essentially boils down to showing that the variance of the service rate (transmission rate) at each queue, $V[r_{i}]$, is higher in the case of single-layer outage approach when compared to the proposed multi-layer approach.

## 5. *ℓ*-State Channel Multi-Access

In this section, we generalize the multi-access encoding and decoding approach outlined in Section 3 from the special case of 2-state channel, {*weak*, *strong*}, to channel with an arbitrary number of states *ℓ*. We denote the channel states by $\left\{\alpha_{1},\ldots ,\alpha_{\ell }\right\}$. Without loss of generality, we assume that $0<\alpha_{1}<\cdots <\alpha_{\ell }<+\infty$. Similarly to Section 2, we consider a slowly fading non-orthogonal multiple access channel model with N-transmitters and one receiver. The channel power gain of each user *i* can randomly take one of ℓ-states, i.e., $h_{i}^{2}\in \left\{\alpha_{1},\ldots ,\alpha_{\ell }\right\}$.

In the layering approach in Section 3.1, we ordered the network state according to the number of users experiencing a *strong* channel state. Subsequently, each user splits its message into 2N layers, and the receiver decodes the layers adapted to the actual network state. Similarly, for the *ℓ*-state channel, we order the combined network state according to the number of users in the network sharing a particular state $\alpha_{j}$ as well as the value of such a state. In particular, a combined network state is degraded with respect to another state if it has a strictly smaller sum-rate capacity. We define the column vector $h\overset{▵}{=}{\left[h_{1}^{2},\ldots ,h_{N}^{2}\right]}^{T}$ as the the combined network state and consider that a network state h to be degraded with respect to network state $\tilde{h}$ if and only if

(25) $${∥h∥}_{1}<{∥\tilde{h}∥}_{1}\;.$$

The motivation of such ordering stems from the fact the condition in (25) indicates the state $\tilde{h}$ allows higher sum-rate capacity in an N-user MAC with full CSIT. In order to overcome the absence of full CSIT at each user, a transmitter splits its message into a finite number of layers, each adapted to the combined network state to avoid complete outages. Similarly to Section 3.1, user *i* encodes an available message using (ℓ−1)N+1 independent random Gaussian codebooks. The codewords of these codebooks are denoted by $U_{jk}^{i}$. For layer $U_{jk}^{i}$, j∈{1,…,ℓ} denotes the channel state of user *i*, that is $h_{i}^{2}=\alpha_{j}$, while k∈{0,…,N−1} denotes the number of users in the network with stronger channel state, i.e., $k=\sum_{i=1}^{N}I\left(h_{i}^{2}>\alpha_{j}\right)$ where I(x) is the indicator function.

According to the layering approach outlined above, the receiver attempts to successively decode up to N((ℓ−1)N+1) depending on the exact combined network state h. In particular, when the actual network state is h, the receiver decodes for each user *i* layer $U_{jk}^{i}$ adapted to network state h in addition to all the layers adapted to all degraded network states $\tilde{h}$ such that (25) is satisfied. The number of layers decoded for user *i* at the receiver increases from network state h to network state $\hat{h}$ either if its own channel state becomes stronger or if the number of users experiencing channels strictly stronger than $h_{i}^{2}$ increases.

Given a network state h, the receiver employs up to *M* stages of successive decoding, where *M* denotes the argument of the strongest channel gain in the network, i.e., $M\overset{▵}{=}\arg{\left\|h\right\|}_{\infty }$. In stage n∈{1,…,M}, the receiver successively decodes up to one layer for each user according to a descending order of the channel states among users. The details of the proposed decoding order for the ℓ-state channel are outlined in Algorithm 2.
**Algorithm 2:** Successive Decoding for ℓ-state channel1:**input**h2:**set**$k_{i}=\sum_{d=1}^{N}I\left(h_{d}^{2}>\alpha_{i}\right),\forall i\in N,\;M\overset{▵}{=}\arg{\left\|h\right\|}_{\infty }$3:**for**m∈{1,…,M}4:   Successively decode $\{U_{mk_{i}}^{i}:h_{i}^{2}\geq \alpha_{m},\;\forall i\in N\}\;.$5:**end for**

We remark that according to the proposed layering approach for the *ℓ*-state channel and decoding approach in Algorithm 2, the total number of layers decoded by the receiver from each user *i* is possibly different in certain network states. Although, one possible generalization of the layering policy in Section 3 is that each user adapts a different encoding layer to each possible combined channel state, which in turn requires each user to encode its message into $\ell^{N}$ layers. However, the computational complexity of the decoding process, in addition to determining the optimal power allocation among layers, is considerable as the number of users *N* grows larger. Therefore, we adopt the outlined layering approach where each user splits its message into N(ℓ−1)+1 layers instead of $\ell^{N}$ layers.

## 6. Numerical Evaluations

In this section, we evaluate the average achievable queuing delay for each user in the MAC channel using the multi-access broadcast approach outlined in Section 3. In particular, we adopt a Monte-Carlo simulation to optimally allocate the transmission power among the encoded layers at each user such that the average queuing delay is minimized. We divide the comparison settings into two main parts according to the arrival process at each queue, where we set the arrival process to be the same among both users in each setting. The first considers deterministic arrivals with value λ. The second one considers the Poisson arrival process. Furthermore, we also consider symmetric and asymmetric channel distributions among users. Throughout this section, we set the channel gains to $\alpha_{1}=0.5$ for the weak channel and $\alpha_{2}=1$ for the strong channel gains. In the symmetric case, we set the channel probability distribution for each user as $p_{1}=p_{2}=0.5$, and in the asymmetric case, we set the probabilities to $p_{1}=0.5$ and $p_{2}=0.1$. In the asymmetric model, user 2 encounters a weak channel with a high probability, i.e., ${\bar{p}}_{2}=0.9$. We set the objective function in this numerical simulation to minimize the sum average delays of users 1 and 2 for the broadcast approach. Subsequently, based on the obtained optimal power distribution among the layers at each user, we evaluate the resulting average delay for the outage approach such that the average rates for each user are equal across both approaches.

**Figure 1:**
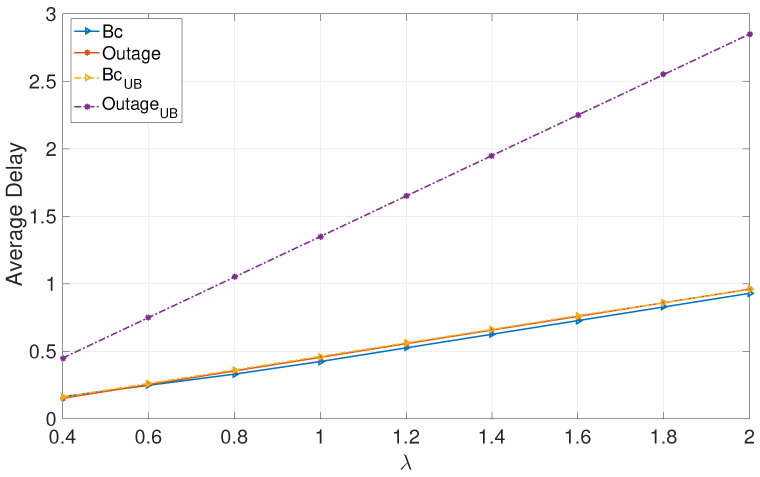
Deterministic: Symmetric.

**Figure 2:**
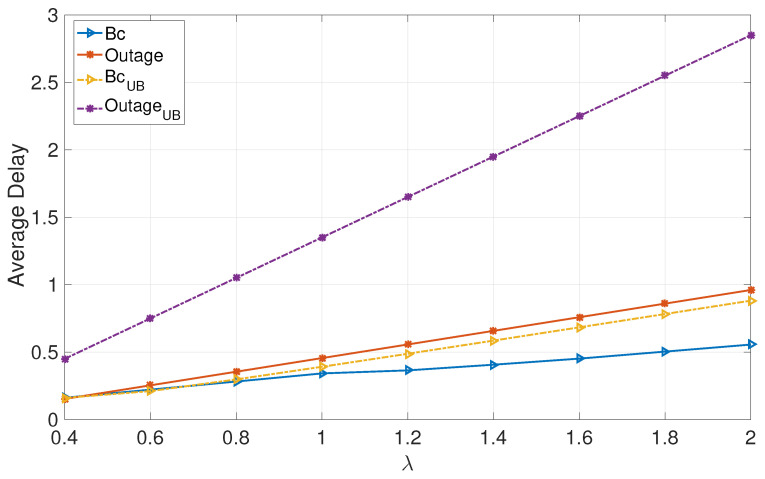
Deterministic: Asymmetric.

**Figure 3:**
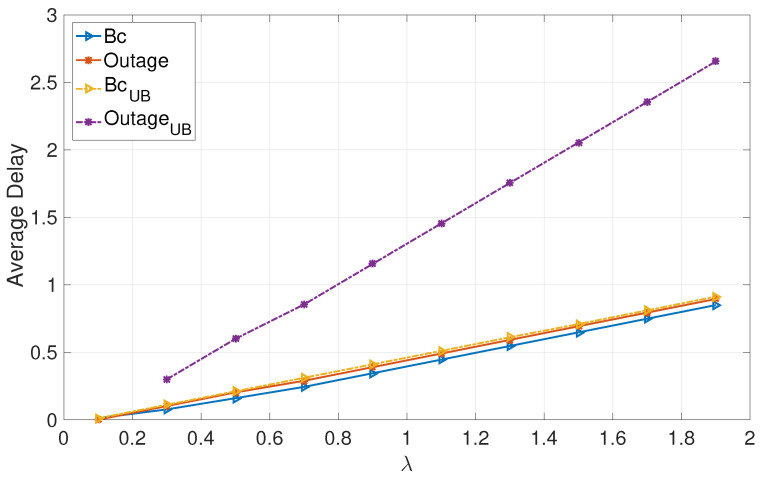
Poisson: Symmetric.

**Figure 4:**
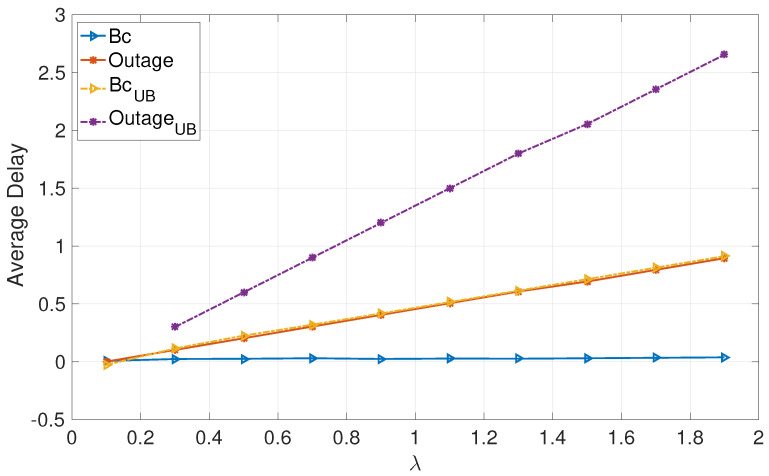
Poisson: Asymmetric.

Figure 1 and Figure 2 focus on deterministic arrivals in the symmetric and asymmetric channel settings. In these figures, we compare average delay versus varying arrival rate λ in the proposed broadcast approach (denoted by “Bc”) and in the outage approach (denoted by “outage”). In these evaluations, we have set the SNR to P=10 dB. Furthermore, in these figures, we provide upper bounds that we have characterized for the broadcast approach (denoted by “Bc${}_{\mathrm{UB}}$”) and the outage approach (denoted by “Outage${}_{\mathrm{UB}}$”). Figure 3 and Figure 4 depict the counterparts of these results for Poisson arrival processes. Finally, it is observed that introducing asymmetry in the models (i.e., unequal probabilities for encountering strong channels) slightly improves the average latency of the broadcast approach, whereas it does not have a notable effect in the outage approach.

The numerical evaluations support the analysis, demonstrating that the proposed broadcast approach significantly enhances the average delays of both users in the moderate and high SNR regimes for moderate and high arrival rates.

## 7. Concluding Remarks

In this paper, a non-orthogonal multi-access broadcast approach is employed, in which each user splits its information stream into a finite number of encoded layers, each adapted to one possible network state, serving as an outage-free low-latency transmission scheme. In particular, the average queuing delay of each user under the proposed multi-access approach is analyzed for different arrival processes at each transmitter. First, for deterministic arrivals, closed-form lower and upper bounds on the average delay are derived analytically. Secondly, for Poisson arrival rates, the average queuing delay is characterized in a closed form. The latency advantage of the proposed approach compared to the single-layer transmission is shown analytically. Finally, we note that in this paper, our focus has been on the discrete channel models since it provides a setting based on which the key ideas (specifically information layering and decoding strategy) can be described clearly and in detail. In order to gain insight into the behavior in the continuous channel models, by increasing the number of channel states in the limit of an infinite number of states, the models converge to a continuous model, and the codebook assignments and decoding strategy converge to their counterparts for continuous channels (larger number of codebooks with low rates).

## Figures and Tables

**Table 1:** Layering and codebook assignments by user *i*.

$h_{i}^{2}$	*k*	0	1	2	⋯	N−1	*N*
$\alpha_{1}$		$U_{10}^{i}$	$U_{11}^{i}$	$U_{12}^{i}$	⋯	$U_{1N-1}^{1}$	
$\alpha_{2}$			$U_{21}^{i}$	$U_{22}^{i}$	⋯	$U_{2N-1}^{i}$	$U_{2N}^{i}$

**Table 2:** Decoded layers for channel state $E_{k}$ where $h_{i}^{2}=\alpha_{j}$.

Stage	Stage 1	Stage 2	Stage 3	Stage 4	…	Stage 4k+1
**Codebook**	$P_{10}$	$Q_{1k}$	$P_{11}$	$Q_{2(k-1)}$	…	$\left\{U_{2k}^{1}:i\in S_{k}\right\}$

## Appendix A. Constants of Theorem 1

∀i∈N:

(A1) $$\begin{array}{cc} d_{10}^{i}\left(\phi \right) & \overset{▵}{=}C\left(\alpha_{1}\beta_{10}^{i},N\alpha_{1}-\sum_{j=1}^{i}\alpha_{1}\beta_{10}^{j}\right)\;. \end{array}$$

$\forall m\in S_{k}:$

(A2) $$\begin{array}{cc} d_{10}^{m}\left(S_{k}\right) & \overset{▵}{=}C\left(\alpha_{2}\beta_{10}^{m},\left(N-k\right)\alpha_{1}+k\alpha_{2}-\sum_{j\in S_{k},j\leq k\left(m\right)}\alpha_{2}\left(1-\beta_{10}^{j}\right)\right)\;, \end{array}$$

(A3) $$\begin{array}{cc} d_{11}^{m}\left(S_{k}\right) & \overset{▵}{=}C\left(\alpha_{2}\beta_{11}^{m},\left(N-k\right)\alpha_{1}+k\alpha_{2}-\sum_{j\in S_{k}}\alpha_{2}\beta_{10}^{j}-\sum_{j\notin S_{k}}\alpha_{1}\beta_{1k}^{j}-\sum_{j\in S_{k},j\leq k\left(m\right)}\alpha_{2}\beta_{11}^{j})\right)\;, \end{array}$$

(A4) $$\begin{array}{cc} d_{21}^{m}\left(S_{k}\right) & \overset{▵}{=}C\left(\alpha_{2}\beta_{21}^{m},\left(N-k\right)\alpha_{1}+k\alpha_{2}-\sum_{j\in S_{k}}\alpha_{2}\left(\beta_{10}^{j}+\beta_{11}^{j}\right)\right.\\ \; & \left.\;\;-\sum_{j\notin S_{k}}\alpha_{1}\left(\beta_{1k}^{j}+\beta_{2(k-1)}^{j}\right)-\sum_{j\in S_{k},j\leq k\left(m\right)}\alpha_{2}\beta_{21}^{j})\right) \end{array}$$

$\forall m\in S_{k}$ and ℓ∈{1,…,k}:

(A5) $$\begin{array}{cc} d_{1\ell }^{m}\left(S_{k}\right) & \overset{▵}{=}C\left(\alpha_{2}\beta_{1\ell }^{m},\left(N-k\right)\alpha_{1}+k\alpha_{2}-\sum_{j\in S_{k}}\alpha_{2}\beta_{10}^{j}-\sum_{j\in S_{k}}\sum_{i=1}^{\ell -1}\alpha_{2}\left(\beta_{1i}^{j}+\beta_{2i}^{j}\right)\right.\\ \; & \left.\;\;-\sum_{j\notin S_{k}}\alpha_{1}\sum_{i=1}^{\ell -1}\left(\beta_{1(k-i+1)}^{j}+\beta_{2(k-i+1)}^{j}\right)-\sum_{j\in S_{k},j\leq k\left(m\right)}\alpha_{2}\beta_{1\ell }^{j}\right)\;, \end{array}$$

(A6) $$\begin{array}{cc} d_{2\ell }^{m}\left(S_{k}\right) & \overset{▵}{=}C\left(\alpha_{2}\beta_{2\ell }^{m},\left(N-k\right)\alpha_{1}+k\alpha_{2}-\sum_{j\in S_{k}}\alpha_{2}\beta_{10}^{j}-\sum_{j\in S_{k}}\sum_{i=1}^{\ell }\alpha_{2}\beta_{1i}^{j}-\sum_{j\in S_{k}}\sum_{i=1}^{\ell -1}\alpha_{2}\beta_{2i}^{j}\right.\\ \; & \left.\;\;-\sum_{j\notin S_{k}}\alpha_{1}\sum_{i=1}^{\ell }\left(\beta_{1(k-i+1)}^{j}+\beta_{2(k-i+1)}^{j}\right)-\sum_{j\in S_{k},j\leq k\left(m\right)}\alpha_{2}\beta_{2\ell }^{j}\right)\;. \end{array}$$

$\forall n\notin S_{k}$ and ℓ∈{1,…,k}:

(A7) $$\begin{array}{cc} d_{1(k-\ell +1)}^{n}\left(S_{k}\right) & ≜C\left(\alpha_{1}\beta_{1(k-\ell +1)}^{n},\left(N-k\right)\alpha_{1}+k\alpha_{2}-\sum_{j\in S_{k}}\alpha_{2}\beta_{10}^{j}-\sum_{j\in S_{k}}\sum_{i=1}^{\ell -1}\alpha_{2}\left(\beta_{1i}^{j}+\beta_{2i}^{j}\right)\right.\\ \; & \left.-\sum_{j\notin S_{k}}\alpha_{1}\sum_{i=1}^{\ell -1}\left(\beta_{1(k-i+1)}^{j}+\beta_{2(k-i+1)}^{j}\right)-\sum_{j\notin S_{k},j\leq \bar{k}\left(n\right)}\alpha_{1}\beta_{1(k-\ell +1)}^{j})\right)\;, \end{array}$$

(A8) $$\begin{array}{cc} d_{2(k-\ell )}^{n}\left(S_{k}\right) & ≜C\left(\alpha_{1}\beta_{2(k-\ell )}^{n},\left(N-k\right)\alpha_{1}+k\alpha_{2}-\sum_{j\in S_{k}}\alpha_{2}\beta_{10}^{j}-\sum_{j\in S_{k}}\sum_{i=1}^{\ell }\alpha_{2}\beta_{1i}^{j}-\sum_{j\in S_{k}}\sum_{i=1}^{\ell -1}\alpha_{2}\beta_{2i}^{j}\right.\\ \; & \left.-\sum_{j\notin S_{k}}\sum_{i=1}^{\ell }\alpha_{1}\beta_{1(k-i+1}^{j}-\sum_{j\notin S_{k}}\sum_{i=1}^{\ell -1}\alpha_{1}\beta_{2(k-i+1)}^{j}-\sum_{j\notin S_{k},j\leq \bar{k}\left(n\right)}\alpha_{1}\beta_{2(k-\ell )}^{j})\right)\;. \end{array}$$

## Appendix B. Proof of Theorem 1

The rate region characterized in Theorem 1 is achievable by employing the layering scheme in Section 3.1 at each transmitter combined with the successive decoding strategy in Algorithm 1. Recall that the maximum rate of codeword $U_{jk}^{i}$, for each user i∈N channel j∈{1,2}, and k∈{0∪N}, is bounded by the minimum achievable rate for that codebook in all combined network states during which it is decoded.

We define S as the set of users’ indices that are experiencing a *strong* states. This set is known to the receiver. Accordingly, we define $S_{k}$ as a realization of *S* that contains exactly *k* users, i.e., *k* users have strong channels and N−k users have weak channels. Next, we discuss $S_{0}$ and $S_{k}$ for k∈N, separately.

**|S|=0:** All channels are weak

In the event of a network state with all channels in the weak state, $h_{i}^{2}=\alpha_{1},\;\forall i\in N$, the receiver decodes only one layer per user. Specifically, it decodes $\left\{U_{10}^{i}:i\in N\right\}$. It performs successive decoding, starting from user 1 and continuing in the ascending order of users’ indices. In order to successfully decode layers $\left\{U_{10}^{i}:i\in N\right\}$, the rate of each layer i∈N should satisfy:

(A9) $$\begin{array}{cc} \forall i\in N:\;R_{10}^{i} & \leq C\left(\alpha_{1}\beta_{10}^{i},N\alpha_{1}-\sum_{j=1}^{i}\alpha_{1}\beta_{10}^{j}\right)\overset{▵}{=}d_{10}^{i}\left(\phi \right). \end{array}$$

Note that the second argument C(x,y) represents the undecoded layers that will be treated as interference for layer $U_{10}^{i}$. Hence, based on the successive decoding procedure, when the receiver decodes $U_{10}^{i}$, layers $U_{10}^{i}$ for users j∈{1,…,i−1} have already been decoded. Thus, their interference is subtracted from the total transmitted signal, accounted by the term $1-\beta_{10}^{j}$. On the other hand, none of the layers transmitted by users j∈{i+1,…,N} have been decoded yet, which is accounted by the term $\left(N-i\right)\alpha_{1}$.

Next, we will characterize upper bounds on the achievable rates of all the layers decoded when there are exactly *k* users with strong channels, i.e., |S|=k.

**|S|=k: ***k* channels are strong

As discussed earlier, when |S|=k, the receiver employs 4k+1 decoding stages. For this purpose, the set of codebooks $\left\{U_{j\ell }^{i}:i\in N\right\}$ is partitioned to two sets

(A10) $$\begin{array}{c} P_{j\ell }≜\left\{U_{j\ell }^{i}:i\in S\right\}\;\mathrm{and}\;Q_{j\ell }≜\left\{U_{j\ell }^{i}:i\notin S\right\}\;, \end{array}$$

rendering a total of 4k+1 partitions for different j∈{1,2} and ℓ∈{0,…,k}. The decoding strategy, decodes one message from each of these, except for the partition $\left\{U_{2k}^{i}:i\notin S\right\}$. The decoding strategy works as follows. We create the following two sequences of sets:

(A11) $$\begin{array}{cc} P & ≜\{P_{10},P_{11},P_{21},\ldots ,P_{2(k-1)},P_{1k},\}\;, \end{array}$$

(A12) $$\begin{array}{cc} Q & ≜\{Q_{1k},Q_{2(k-1)},Q_{1(k-1)},\ldots ,Q_{11},Q_{10},\}\;. \end{array}$$

The decoding strategy selects codebooks by alternating between P and Q an an ascending order and decodes exactly one codebook from each. This results in 4k coding stages. Finally, the codebooks in $\left\{U_{2k}^{i}:i\in S\right\}$ are decoded as the last stage, i.e., stage 4k+1.

- **Decoding stage 1:**
  We start by decoding the layers $P_{10}≜\left\{U_{10}^{i}:i\in S\right\}$. Recall that $S_{k}$ was defined as an ordered set of these users. The codebooks will be decoded sequentially in this order. When $m\in S_{k}$, we denote the position of *m* in $S_{k}$ by k(m). Hence, $\forall m\in S_{k}$
  (A13) $$\begin{array}{cc} R_{10}^{m} & \leq C\left(\alpha_{2}\beta_{10}^{m},\left(N-k\right)\alpha_{1}+k\alpha_{2}-\sum_{j\in S_{k},j\leq k\left(m\right)}\alpha_{2}\left(1-\beta_{10}^{j}\right)\right)\overset{▵}{=}d_{10}^{m}\left(S_{k}\right)\;. \end{array}$$
- **Decoding stage 2:**
  Next, we sequentially decode the layers in $Q_{1k}=\left\{U_{1k}^{i}:i\notin S\right\}$, which involves layers $U_{1k}^{i}$ of users with weak channels. When $n\notin S_{k}$, we denote the position of *n* in the ordered set $N\backslash S_{k}$ by $\bar{k}\left(n\right)$. Hence, $\forall n\notin S_{k}$
  (A14) $$\begin{array}{cc} R_{1k}^{n} & \leq C\left(\alpha_{1}\beta_{1k}^{n},\left(N-k\right)\alpha_{1}+k\alpha_{2}-\sum_{j\in S_{k}}\alpha_{2}\beta_{10}^{j}-\sum_{j\notin S_{k},j<\bar{k}\left(n\right)}\alpha_{1}\beta_{1k}^{j}\right)\overset{▵}{=}d_{1k}^{n}\left(S_{k}\right)\;. \end{array}$$
- **Decoding stage 3:**
  In the third stage, the codebooks in $P_{10}$ and $Q_{1k}$ are already decoded. We continue by sequentially decoding the set of codebooks in $P_{11}≜\left\{U_{11}^{i}:i\in S\right\}$. Hence, $\forall m\in S_{k}$
  (A15) $$\begin{array}{cc} R_{11}^{m} & \leq C\left(\alpha_{2}\beta_{11}^{m},\left(N-k\right)\alpha_{1}+k\alpha_{2}-\sum_{j\in S_{k}}\alpha_{2}\beta_{10}^{j}-\sum_{j\notin S_{k}}\alpha_{1}\beta_{1k}^{j}-\sum_{j\in S_{k},j\leq k\left(m\right)}\alpha_{2}\beta_{11}^{j})\right)\overset{▵}{=}d_{11}^{m}\left(S_{k}\right)\;. \end{array}$$
- **Decoding stage 4:**
  The decoding process continues by sequentially decoding the codebooks in $Q_{2(k-1)}=\left\{U_{2(k-1)}^{i}:i\notin S\right\}$, while the codebooks of $P_{10}$, $Q_{1k}$, and $P_{11}$ are already decoded. Hence, for $n\notin S_{k}$
  (A16) $$\begin{array}{cc} R_{2(k-1)}^{n} & \leq C\left(\alpha_{1}\beta_{2(k-1)}^{n},\left(N-k\right)\alpha_{1}+k\alpha_{2}-\sum_{j\in S_{k}}\alpha_{2}\left(\beta_{10}^{j}+\beta_{11}^{j}\right)\right.\\ \; & \left.-\sum_{j\notin S_{k}}\alpha_{1}\beta_{1k}^{j}-\sum_{j\notin S_{k},j\leq \bar{k}\left(n\right)}\alpha_{1}\beta_{2(k-1)}^{j})\right)\overset{▵}{=}d_{2(k-1)}^{n}\left(S_{k}\right)\;. \end{array}$$
- **Decoding stage 5:**
  This stage sequentially decodes the codebooks $P_{21}$. For all $m\in S_{k}^{i}$ we have
  (A17) $$\begin{array}{cc} R_{21}^{m} & \leq C\left(\alpha_{2}\beta_{21}^{m},\left(N-k\right)\alpha_{1}+k\alpha_{2}-\sum_{j\in S_{k}}\alpha_{2}\left(\beta_{10}^{j}+\beta_{11}^{j}\right)\right.\\ \; & \left.-\sum_{j\notin S_{k}}\alpha_{1}\left(\beta_{1k}^{j}+\beta_{2(k-1)}^{j}\right)-\sum_{j\in S_{k},j\leq k\left(m\right)}\alpha_{2}\beta_{21}^{j})\right)\overset{▵}{=}d_{21}^{m}\left(S_{k}\right)\;. \end{array}$$
- **Decoding stage 6:**
  This stage sequentially decodes the codebooks in $Q_{1(k-1)}$. Hence, $\forall n\notin S_{k}$ we have
  (A18) $$\begin{array}{cc} R_{1(k-1)}^{n} & \leq C\left(\alpha_{1}\beta_{1(k-1)}^{n},\left(N-k\right)\alpha_{1}+k\alpha_{2}-\sum_{j\in S_{k}}\alpha_{2}\left(\beta_{10}^{j}+\beta_{11}^{j}+\beta_{21}^{j}\right)\right.\\ \; & \left.-\sum_{j\notin S_{k}}\alpha_{1}\left(\beta_{1k}^{j}+\beta_{2(k-1)}^{j}\right)-\sum_{j\notin S_{k},j\leq \bar{k}\left(n\right)}\alpha_{1}\beta_{1(k-1)}^{j})\right)\overset{▵}{=}d_{1(k-1)}^{n}\left(S_{k}\right)\;. \end{array}$$
- **Decoding stages {2,…,4k+1}:**
  Following the pattern of the previous decoding stages, in general in the stage {2,…,4k}, we decode the codebooks according to the following schedule, for ℓ∈{1,…,k}:
  (A19) $$\begin{array}{c} \begin{array}{cc} \mathrm{codebooks}\mathrm{in}Q_{1(k-\ell +1)} & \;\mathrm{stage}\;4\ell -2\\ \mathrm{codebooks}\mathrm{in}P_{1\ell } & \;\mathrm{stage}\;4\ell -1\\ \mathrm{codebooks}\mathrm{in}Q_{2(k-\ell )} & \;\mathrm{stage}\;4\ell \\ \mathrm{codebooks}\mathrm{in}P_{2\ell } & \;\mathrm{stage}\;4\ell +1 \end{array} \end{array}$$
  Accordingly, we obtain the following rate constraints.
- **Decoding stage 4ℓ−2:**
  By sequentially decoding the messages in $Q_{1(k-\ell +1)}$, $\forall n\notin S_{k}$ we have
  (A20) $$\begin{array}{cc} R_{1(k-\ell +1)}^{n} & \leq C\left(\alpha_{1}\beta_{1(k-\ell +1)}^{n},\left(N-k\right)\alpha_{1}+k\alpha_{2}-\sum_{j\in S_{k}}\alpha_{2}\beta_{10}^{j}-\sum_{j\in S_{k}}\sum_{i=1}^{\ell -1}\alpha_{2}\left(\beta_{1i}^{j}+\beta_{2i}^{j}\right)\right.\\ \; & \left.-\sum_{j\notin S_{k}}\alpha_{1}\sum_{i=1}^{\ell -1}\left(\beta_{1(k-i+1)}^{j}+\beta_{2(k-i+1)}^{j}\right)-\sum_{j\notin S_{k},j\leq \bar{k}\left(n\right)}\alpha_{1}\beta_{1(k-\ell +1)}^{j})\right)\overset{▵}{=}d_{1(k-\ell +1)}^{n}\left(S_{k}\right)\;. \end{array}$$
- **Decoding stage 4ℓ−1:**
  By sequentially decoding the messages in $P_{1\ell }$, $\forall m\in S_{k}$ we have
  (A21) $$\begin{array}{cc} R_{1\ell }^{m} & \leq C\left(\alpha_{2}\beta_{1\ell }^{m},\left(N-k\right)\alpha_{1}+k\alpha_{2}-\sum_{j\in S_{k}}\alpha_{2}\beta_{10}^{j}-\sum_{j\in S_{k}}\sum_{i=1}^{\ell -1}\alpha_{2}\left(\beta_{1i}^{j}+\beta_{2i}^{j}\right)\right.\\ \; & \left.-\sum_{j\notin S_{k}}\alpha_{1}\sum_{i=1}^{\ell -1}\left(\beta_{1(k-i+1)}^{j}+\beta_{2(k-i+1)}^{j}\right)-\sum_{j\in S_{k},j\leq k\left(m\right)}\alpha_{2}\beta_{1\ell }^{j}\right)\overset{▵}{=}d_{1\ell }^{m}\left(S_{k}\right)\;. \end{array}$$
- **Decoding stage 4ℓ:**
  By sequentially decoding the messages in $Q_{1(k-\ell )}$, $\forall n\notin S_{k}$ we have
  (A22) $$\begin{array}{cc} R_{2(k-\ell )}^{n} & \leq C\left(\alpha_{1}\beta_{2(k-\ell )}^{n},\left(N-k\right)\alpha_{1}+k\alpha_{2}-\sum_{j\in S_{k}}\alpha_{2}\beta_{10}^{j}-\sum_{j\in S_{k}}\sum_{i=1}^{\ell }\alpha_{2}\beta_{1i}^{j}-\sum_{j\in S_{k}}\sum_{i=1}^{\ell -1}\alpha_{2}\beta_{2i}^{j}\right.\\ \; & \left.-\sum_{j\notin S_{k}}\sum_{i=1}^{\ell }\alpha_{1}\beta_{1(k-i+1}^{j}-\sum_{j\notin S_{k}}\sum_{i=1}^{\ell -1}\alpha_{1}\beta_{2(k-i+1)}^{j}-\sum_{j\notin S_{k},j\leq \bar{k}\left(n\right)}\alpha_{1}\beta_{2(k-\ell )}^{j})\right)\overset{▵}{=}d_{2(k-\ell )}^{n}\left(S_{k}\right)\;. \end{array}$$
- **Decoding stage 4ℓ+1:**
  By sequentially decoding the messages in $P_{2\ell }$, $\forall m\in S_{k}$ we have
  (A23) $$\begin{array}{cc} R_{2\ell }^{m} & \leq C\left(\alpha_{2}\beta_{2\ell }^{m},\left(N-k\right)\alpha_{1}+k\alpha_{2}-\sum_{j\in S_{k}}\alpha_{2}\beta_{10}^{j}-\sum_{j\in S_{k}}\sum_{i=1}^{\ell }\alpha_{2}\beta_{1i}^{j}-\sum_{j\in S_{k}}\sum_{i=1}^{\ell -1}\alpha_{2}\beta_{2i}^{j}\right.\\ \; & \left.-\sum_{j\notin S_{k}}\alpha_{1}\sum_{i=1}^{\ell }\left(\beta_{1(k-i+1)}^{j}+\beta_{2(k-i+1)}^{j}\right)-\sum_{j\in S_{k},j\leq k\left(m\right)}\alpha_{2}\beta_{2\ell }^{j}\right)\overset{▵}{=}d_{2\ell }^{m}\left(S_{k}\right)\;. \end{array}$$

Given the upper bounds on the individual achievable rates of $U_{jk}^{i},\forall i\in N,j\in 1,2,k\in \left\{0\cup N\right\}$, the maximum achievable rate of $U_{jk}^{i}$ is bounded my the minimum upper bound among all the network states within which it is decoded.

## Appendix C. Proof of Theorem 2

By applying a change of variable to each term and taking the integral $\int_{0}^{\infty }e^{-sq}dF_{1}\left(q\right)$ as a common factor, $L_{1}\left(s\right)$ can be expressed as

(A24) $$\begin{array}{cc} L_{1}\left(s\right) & =\frac{F_{1}\left(0\right)-\int_{0^{+}}^{\left(\sum_{ij}R_{ij}^{1}-\lambda_{1}\right)}e^{-s(q+\left(\lambda_{1}-\sum_{ij}R_{ij}^{1}\right))}dF_{1}\left(q\right)}{1-[{\bar{p}}_{1}{\bar{p}}_{2}e^{-s(\lambda_{1}-\sum_{ij}R_{ij}^{1})}+{\bar{p}}_{1}p_{2}e^{-s(\lambda_{1}-R_{11}^{1}-R_{21}^{1}))}+p_{1}{\bar{p}}_{2}e^{-s(\lambda_{1}-R_{11}^{1}-R_{12}^{1}))}+p_{1}p_{2}e^{-s(\lambda_{1}-R_{11}^{1}))}]} \end{array}$$

Further, by using the definition of $F_{1}\left(0\right)={\bar{p}}_{1}{\bar{p}}_{2}F_{1}\left(q-\left(\lambda_{1}-\sum_{ij}R_{ij}^{1}\right)\right)$ and multiplying the numerator and denominator of (A24) by a common factor, $e^{-s(\sum_{ij}R_{ij}^{1}-\lambda_{1})}$, we have.

(A25) $$\begin{array}{c} L_{1}\left(s\right)=\frac{{\bar{p}}_{1}{\bar{p}}_{2}\left[\int_{0}^{\left(\sum_{ij}R_{ij}^{1}-\lambda_{1}\right)}e^{-s(\sum_{ij}R_{ij}^{1}-\lambda_{1})}-e^{-sq}dF_{1}\left(q\right)\right]}{e^{-s(\sum_{ij}R_{ij}^{1}-\lambda_{1})}-\left[{\bar{p}}_{1}{\bar{p}}_{2}+{\bar{p}}_{1}p_{2}e^{-s(R_{12}^{1}+R_{22}^{1}))}+p_{1}{\bar{p}}_{2}e^{-s(R_{21}^{1}+R_{22}^{1}))}+p_{1}p_{2}e^{-s(R_{21}^{1}+R_{12}^{1}+R_{22}^{1}))}\right]}\;\\ \;\;\;\overset{▵}{=}\frac{D_{1}\left(s\right)}{N_{1}\left(s\right)}\;. \end{array}$$

It can be readily noticed from (A25) that $\lim_{s\to 0}D_{1}\left(s\right)=\lim_{s\to 0}N_{1}\left(s\right)=0$, therefore we apply L’hopital’s limit rule on (A25) to arrive at

(A26) $$\begin{array}{c} E\left[Q_{1}\right]=\lim_{s\to 0}\frac{D_{Q_{1}}^{{}^{\prime \prime }}\left(s\right)-N_{Q_{1}}^{{}^{\prime \prime }}\left(s\right)}{2D_{Q_{1}}^{{}^{\prime }}\left(s\right)}\;. \end{array}$$

Finally, we evaluate the terms $D_{Q_{1}}^{{}^{\prime \prime }}\left(s\right)$, $N_{Q_{1}}^{{}^{\prime \prime }}\left(s\right)$ and $D_{Q_{1}}^{{}^{\prime }}\left(s\right)$ where we have

(A27) $$\begin{array}{cc} \lim_{s\to 0}D_{Q_{1}}^{{}^{\prime }}\left(s\right) & =-\left(\sum_{ij}R_{ij}^{1}-\lambda_{1}\right)+{\bar{p}}_{1}p_{2}\left(R_{12}^{1}+R_{22}^{1}\right)+p_{1}{\bar{p}}_{2}\left(R_{12}^{1}+R_{22}^{1}\right)+p_{1}p_{2}\left(R_{12}^{1}+R_{21}^{1}+R_{22}^{1}\right)\; \end{array}$$

(A28) $$\begin{array}{cc} \lim_{s\to 0}D_{Q_{1}}^{{}^{\prime \prime }}\left(s\right) & ={\left(\sum_{ij}R_{ij}^{1}-\lambda_{1}\right)}^{2}-{\bar{p}}_{1}p_{2}{\left(R_{12}^{1}+R_{22}^{1}\right)}^{2}-p_{1}{\bar{p}}_{2}{\left(R_{12}^{1}+R_{22}^{1}\right)}^{2}-p_{1}p_{2}{\left(R_{12}^{1}+R_{21}^{1}+R_{22}^{1}\right)}^{2}\;, \end{array}$$

and

(A29) $$\begin{array}{cc} \lim_{s\to 0}N_{Q_{1}}^{{}^{\prime \prime }}\left(s\right) & ={\bar{p}}_{1}{\bar{p}}_{2}\int_{0}^{\left(\sum_{ij}R_{ij}^{1}-\lambda_{1}\right)}\left[{\left(\sum_{ij}R_{ij}^{1}-\lambda_{1}\right)}^{2}-q^{2}\right]dF_{1}\left(q\right)\;. \end{array}$$

Finally, by using $\lim_{s\to 0}D_{1}^{{}^{\prime }}\left(s\right)=\lim_{s\to 0}N_{1}^{{}^{\prime }}\left(s\right)$, the second derivative of the numerator term can be upper bounded by replacing $\left(\sum_{ij}R_{ij}^{1}-\lambda_{1}+q\right)$ by $2(\sum_{ij}R_{ij}^{1}-\lambda_{1})$ arriving at

(A30) $$\begin{array}{cc} \lim_{s\to 0}N_{Q_{1}}^{{}^{\prime \prime }}\left(s\right) & \leq 2\left(\sum_{ij}R_{ij}^{1}-\lambda_{1}\right)\left(\sum_{ij}R_{ij}^{1}-\lambda_{1}\right.\\ \; & \left.-{\bar{p}}_{1}p_{2}\left(R_{12}^{1}+R_{22}^{1}\right)-p_{1}{\bar{p}}_{2}\left(R_{12}^{1}+R_{22}^{1}\right)-p_{1}p_{2}\left(R_{12}^{1}+R_{21}^{1}+R_{22}^{1}\right)\right)\;. \end{array}$$

Next, we leverage (A26) reaching

(A31) $$\begin{array}{cc} \; & E\left[Q_{i}\right]\geq \frac{1}{2}\sum_{j=1}^{2}\sum_{k=0}^{N}R_{jk}^{i}-\frac{\lambda_{i}}{2}-\frac{N_{i}}{D_{i}}\;,\\ \; & E\left[Q_{i}\right]\leq \sum_{j=1}^{2}\sum_{k=0}^{N}R_{jk}^{i}-\lambda_{i}-\frac{N_{i}}{D_{i}}\;, \end{array}$$

where

(A32) $$\begin{array}{cc} \; & N_{i}\overset{▵}{=}-{\left(\sum_{j=1}^{2}\sum_{k=0}^{N}R_{jk}^{i}-\lambda_{i}\right)}^{2}\\ \; & +P\left[E\left({\bar{S}}_{0}^{i}\right)\right]{\left(\sum_{j=1}^{2}\sum_{k=1}^{N}R_{jk}^{i}\right)}^{2}\\ \; & +\sum_{\ell =1}^{N-1}P\left[E\left(S_{\ell }^{i}\right)\right]\cdot {\left(\sum_{j=1}^{2}\sum_{k=\ell +1}^{N}R_{jk}^{i}+R_{1\ell }^{i}\right)}^{2}\\ \; & +\sum_{\ell =1}^{N-1}P\left[E\left({\bar{S^{i}}}_{\ell }\right)\right]\cdot {\left(\sum_{j=1}^{2}\sum_{k=\ell +1}^{N}R_{jk}^{i}+R_{2\ell }^{i}\right)}^{2}\\ \; & D_{i}\overset{▵}{=}2\left(E\left[r_{i}\right]-\lambda_{i}\right)\;. \end{array}$$

## Appendix D. Proof of Theorem 3

In this Appendix, we base the proof of Theorem 3 on two main steps. First, we characterize a lower bound on the average achievable rate of each user *i* using a single layer per user (outage approach). Secondly, we derive the rate of increase of the average achievable delay with respect to the average arrival rate $\lambda_{i}$ (first-order derivative) for the delay upper bound of the multi-layer approach to that of the delay lower bound of the outage approach. Finally, under a fixed average achievable rate among both approaches, we show that the proposed approach outperforms the single layer outage approach.

Recalling the recursive expression for $Q_{i}$ in terms of the variable $Z_{i}$ in (3), a recursive form of $F_{i}\left(q\right)$ can be expressed as follows [52,53]

(A33) $$\begin{array}{c} F_{i}\left(q\right)=\left\{\begin{array}{cc} 0, & \;q<0\\ \int_{-\infty }^{q}F_{i}\left(q-\tau \right)dF_{Z_{i}}\left(\tau \right), & \;q\geq 0\;, \end{array}\right. \end{array}$$

where $dF_{Z_{i}}\left(z\right)$ denote pdf of $Z_{i}$.

At the end of every transmission block, the change in queue size *i*, $Z_{i}$, is primarily determined by the difference between the data arrival $\lambda_{i}$ and the fixed rate successfully decoded at the receiver, which in turn is determined by the combined network state. Consequently, $dF_{Z_{i}}\left(z\right)$ can be expressed by

(A34) $$\begin{array}{cc} dF_{Z_{i}}\left(z\right) & =P_{\mathrm{out}}\delta \left(z-\lambda_{i}+R_{F}\right)\;. \end{array}$$

We remark that in order to guarantee the stability of every queue *i*, we assume that the arrival rate $\lambda_{i}$ is less that the average achievable rate (service rate of the queue), i.e.,

(A35) $$\begin{array}{c} \lambda_{i}<P_{\mathrm{out}}R_{F},\;\forall i\in N\;. \end{array}$$

Combining (12) and (13), an explicit expression for $F_{i}\left(q\right),\forall i\in N$ is given by

(A36) $$\begin{array}{cc} \; & F_{i}\left(q\right)=\\ \; & \left\{\begin{array}{cc} 0\;, & \;\forall q<0\\ P_{\mathrm{out}}F_{i}\left(q-\lambda_{i}+R_{F}\right)\;, & \;\forall q\geq 0 \end{array}\right.\;. \end{array}$$

Finally, we evaluate the terms $D_{Q_{1}}^{{}^{\prime \prime }}\left(s\right)$ and $N_{Q_{1}}^{{}^{\prime \prime }}\left(s\right)$ where we have

(A37) $$\begin{array}{cc} \lim_{s\to 0}D_{Q_{1}}^{{}^{\prime \prime }}\left(s\right) & ={\left(R_{F}-\lambda_{i}\right)}^{2}-\left(1-P_{\mathrm{out}}\right)R_{F}^{2}\;, \end{array}$$

and

(A38) $$\begin{array}{cc} \lim_{s\to 0}N_{Q_{1}}^{{}^{\prime \prime }}\left(s\right) & =P_{\mathrm{out}}\int_{0}^{R_{F}-\lambda_{1})}\left[{\left(R_{F}-\lambda_{1}\right)}^{2}-q^{2}\right]dF_{1}\left(q\right)\;. \end{array}$$

Finally, by using $\lim_{s\to 0}D_{1}^{{}^{\prime }}\left(s\right)=\lim_{s\to 0}N_{1}^{{}^{\prime }}\left(s\right)$, the second derivative of the numerator term can be lower bounded by replacing $\left(F_{F}-\lambda_{1}+q\right)$ by $\left(R_{F}-\lambda_{1}\right)$ arriving at

(A39) $$\begin{array}{cc} \lim_{s\to 0}N_{Q_{1}}^{{}^{\prime \prime }}\left(s\right) & \geq \left(R_{F}-\lambda_{1}\right)\left(R_{F}-\lambda_{1}-P_{\mathrm{out}}R_{F}\right)\;. \end{array}$$

and substitute (A26) reaching

(A40) $$\begin{array}{cc} \; & E\left[Q_{i}\right]\geq \frac{1}{2}R_{F}-\frac{\lambda_{i}}{2}-\frac{N_{i}}{D_{i}}\;, \end{array}$$

where

(A41) $$\begin{array}{cc} \; & N_{i}\overset{▵}{=}-{\left(R_{F}-\lambda_{i}\right)}^{2}+\left(1-P_{\mathrm{out}}\right)R_{F}^{2}\\ \; & D_{i}\overset{▵}{=}P_{\mathrm{out}}R_{F}-\lambda_{i}\;. \end{array}$$

By taking the derivative of the upper/lower bounds derived above we reach

(A42) $$\begin{array}{cc} \frac{\partial U_{B}}{\partial \lambda_{i}} & =-1-\frac{\sum_{j=1}^{2}\sum_{k=0}^{N}R_{jk}^{i}-\lambda_{i}}{E\left[r_{i}\right]-\lambda_{i}}-2\frac{N_{i}}{D_{i}^{2}}\;, \end{array}$$

(A43) $$\begin{array}{cc} \frac{\partial L_{B}}{\partial \lambda_{i}} & =-1-\frac{R_{F}-\lambda_{i}}{P_{\mathrm{out}}R_{F}-\lambda_{i}}-2\frac{-{\left(R_{F}-\lambda_{i}\right)}^{2}+\left(1-P_{\mathrm{out}}\right)R_{F}^{2}}{P_{\mathrm{out}}R_{F}-\lambda_{i}}\;. \end{array}$$
